# The emerging postural instability phenotype in idiopathic Parkinson disease

**DOI:** 10.1038/s41531-022-00287-x

**Published:** 2022-03-18

**Authors:** Frank M. Skidmore, William S. Monroe, Christopher P. Hurt, Anthony P. Nicholas, Adam Gerstenecker, Thomas Anthony, Leon Jololian, Gary Cutter, Adil Bashir, Thomas Denny, David Standaert, Elizabeth A. Disbrow

**Affiliations:** 1 Department of Neurology, University of Alabama at Birmingham, Birmingham, AL USA; 2 Department of Electrical & Computer Engineering, University of Alabama at Birmingham, Birmingham, AL USA; 3 Department of Materials Science and Engineering, University of Alabama at Birmingham, Birmingham, AL USA; 4 School of Public Health, Department of Biostatistics, University of Alabama at Birmingham, Birmingham, AL USA; 5 Department of Physical Therapy, University of Alabama at Birmingham, Birmingham, AL USA; 6 Department of Computer and Electrical Engineering, Auburn University, Auburn, AL USA; 7 Department of Neurology, Center for Brain Health, Louisiana State University Health Sciences Center, Shreveport, LA USA

**Keywords:** Parkinson's disease, Parkinson's disease

## Abstract

Identification of individuals at high risk for rapid progression of motor and cognitive signs in Parkinson disease (PD) is clinically significant. Postural instability and gait dysfunction (PIGD) are associated with greater motor and cognitive deterioration. We examined the relationship between baseline clinical factors and the development of postural instability using 5-year longitudinal de-novo idiopathic data (*n* = 301) from the Parkinson’s Progressive Markers Initiative (PPMI). Logistic regression analysis revealed baseline features associated with future postural instability, and we designated this cohort the emerging postural instability (ePI) phenotype. We evaluated the resulting ePI phenotype rating scale validity in two held-out populations which showed a significantly higher risk of postural instability. Emerging PI phenotype was identified before onset of postural instability in 289 of 301 paired comparisons, with a median progression time of 972 days. Baseline cognitive performance was similar but declined more rapidly in ePI phenotype. We provide an ePI phenotype rating scale (ePIRS) for evaluation of individual risk at baseline for progression to postural instability.

## Introduction

One long-recognized transition point in the development of Parkinson disease (PD)-related disability is the development of postural instability. The relationship between disability, gait and balance is frequently examined through the prism of the Hoehn and Yahr (HY) scale^1,2^, the first widely used PD severity scale. Severity was graded using the following criteria: (1) unilateral involvement with minimal or no functional disability; (2) bilateral or midline involvement without impairment of balance; (3) bilateral disease with mild to moderate disability, impaired postural reflexes, physically independent; (4) severely disabling disease, still able to walk unassisted; (5) confinement to a bed or wheelchair unless aided. Jankovic and colleagues later developed a postural instability/gait dysfunction (PIGD) score^3–5^, derived from the Unified Parkinson’s Disease Rating Scale (UPDRS)^6,7^, a widely used structured history and clinical exam.

PIGD includes the inability to execute postural modification in response to changing support conditions, as well as parkinsonian gait characterized by stooped posture, decreased arm swing and shuffling gait. This PIGD phenotype has been associated with disability^8–10^, including rapid progression of both cognitive^11–15^, and motor dysfunction^4,5,12,16,17^. Deficits in measures of global cognition, processing speed, memory, attention and verbal fluency^18^ (for review see refs. ^11,14^) are associated with the PIGD phenotype, as well as balance disturbance^19^, recurrent falls^20,21^, and risk of institutionalization and death^22–24^. Furthermore, people with PIGD-dominant disease show greater motor and intellectual impairment compared to those with the tremor dominant phenotype^3,16,25^. The tremor dominant phenotype is associated with more benign disease and slower disease progression^26^. Haaxma and colleagues^26^ showed that de novo patients initially presenting with tremor had a mean disease onset that was 3.6 years later and showed a 38% slower increase in motor dysfunction (UPDRS III score) compared to those presenting with bradykinesia-rigidity. Taylor and colleagues^27^ found that rate of cognitive decline in MMSE score was 1.5 points higher in the PIGD compared to the tremor-dominant group over 3 years, though baseline motor and cognitive measures were similar across groups.

The PIGD phenotype is also more common in demented versus non-demented people with PD. Alves and colleagues found that of the 61 patients identified with dementia at study onset, 60 had clinically significant gait and balance disturbance, findings which are consistent with a single disease mechanism. Furthermore, using a repeated measures design over an 8-year observation period, Alves and colleagues^12^ found that, compared to patients with a persistent tremor dominant characterization, people who transitioned from tremor to PIGD type had a 56.7 odds ratio for the development of Parkinson disease dementia (PDD), while the odds ratio for persistent PIGD subtype was 80. Thus disease progression also plays a role in phenotype designation^28^. The PIGD cohort had longer disease duration and more severe PD signs than the tremor dominant and indeterminant groups, though there were no differences in age or dopamine dose across categories. Furthermore, 37% of the people in the tremor dominant group transitioned to PIGD in the first four years, with another 35% transitioning in the second four years. Patients that transitioned to PIGD were only diagnosed with dementia after the transition. In contrast, only 4% of participants transitioned from PIGD to tremor dominant categorization^12^. This sequential progression is consistent with the proposed spread of disease from the brain stem to upper brain areas^29^, and individuals with the PIGD phenotype are at high risk of developing diffuse Lewy body disease^2,16,30^. However 14% of the original tremor dominant sample had not transitioned to the PIGD group even after 8 years, demonstrating significant variability in rate of progression.

A current, critical need in the field is to develop methods for early identification of individuals at risk for rapid disease progression, such as the PIGD subgroup, given its relationship to significant cognitive sequelae and widespread Lewy body disease. One possibility is the development of a behavior-based surrogate marker^31^. The MDS UPDRS is a multidimensional scale based on clinical evaluation and patient self-report that has been shown to accurately track H&Y stage and 5-year disease progression^32^. However, both the PD PIGD phenotype and Hoehn and Yahr classification are based on clinical parameters in later stages of disease^5–7^, and are unstable in early disease^33,34^. We propose to fill this gap by developing a risk profile to predict transition to H &Y stage 3 postural instability using individual baseline UPDRS data.

Early recognition of PD patients at high risk for rapid progression is critical for neuroprotective intervention^33^. Early identification of individuals at risk of progression to postural instability, in particular serves two additional goals. First, development of postural instability is associated with global progression of disease, and accordingly identification of at-risk individuals in this context also identifies individuals at higher risk of cognitive progression and disability. Second, selecting individuals with more rapid motor progression will improve the ability to detect the impact of potential disease modifying agents. Specifically, while medication has an impact on gait in PD, in the PIGD phenotype gait can be less responsive to dopamine replacement. Clinical trials design in PD is impacted by the problem of disentangling the acute motor and cognitive response to dopamine replacement from disease progression^34,35^. More effective identification of a phenotype predisposed to a rapid progression may mitigate some of these trial design issues. Such a classification would increase sample homogeneity and reduce statistical noise, providing the potential for a cleaner signal of presence (or absence) of a disease modifying effect.

We accordingly set out to determine if there are clinical features that comprise a “risk phenotype” for postural instability that is detectable at baseline. We developed a risk profile in a derivation set and evaluated the validity of this measure in two validation sets. To operationalize our findings, we labeled our provisional phenotype the “emerging postural instability (ePI) phenotype” and present our results as a clinical scale, the emerging postural instability rating scale (ePIRS).

## Results

### Sample characteristics

**Fig. 1:**
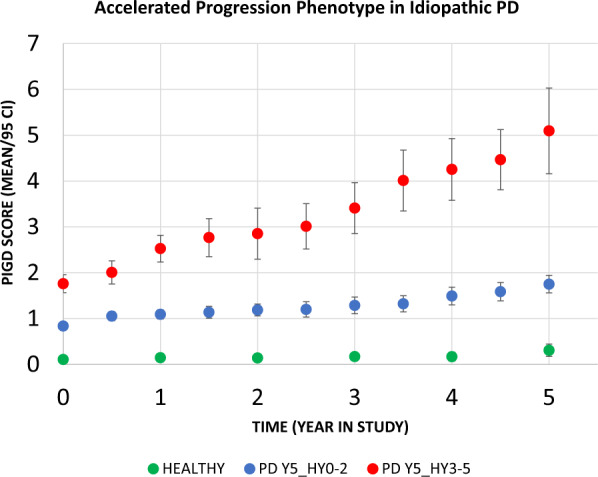
Individuals who had at least one rating of HY 3, on medication, within the first 5 years of PPMI follow up (red circles) were characterized by significantly more rapid progression of gait dysfunction as measured by the PIGD scale compared to individuals who maintained HY status of 2 or less during the first 5 years of treatment (blue circles). Controls (green) are presented as a reference.

In the de-novo cohorts (*n* = 301 for derivation cohort (dIPD) and 79 for validation cohort (vIPD)), the PD sample was demographically similar to the heathy control sample. PD subjects progressing to H&Y scale score >2 by year 5 (Y5_HY3–5) were older and had more severe signs and symptoms at entry (Table 1) and were characterized by progressive gait dysfunction over time as measured by PIGD rating (Fig. 1).

**Table 1:** Median and range of demographic, motor and cognitive measures including sex subgroups.

Measure	Controls	Parkinson disease		Chi-square (LR) (Y5_HY0–2 vs. Y5_HY3–5)
		Y5_HY0–2	Y5_HY3–5	
*Demographics*				
Gender (M, F, Other)	127,62,10	187,73,17	66,33,4	NS
Age	62 (55,69)	61 (53,67)	*67 (59,78)*	***p*** < 0.001**
*Motor examination*				
UPDRS I	2 (0,4)	*3 (1,5)*	*(3,8)*	***p*** < 0.001**
UPDRS II	0 (0,0)	*4 (2,6)*	*7 (4,11)*	***p*** < 0.001**
UPDRS III	0 (0,2)	*18 (13,24)*	*22 (16,29)*	***p*** = 0.0029**
UPDRS IV	N/A	N/A	N/A	
PIGD score	0 (0,0)	*2 (1,3)*	*4 (2,7)*	***p*** < 0.001**
HY	0 (0,0)	*0 (1,2)*	*2 (1,2)*	NS
*Cognition*				
MOCA	28 (27,29)	*28 (26,29)*	*27 (25,29)*	NS
HVLT total recall	26 (23,30)	26 (23,29)	*24 (20,28)*	NS
Delayed recall	10 (8,11)	*9 (8,11)*	*8 (6,10)*	NS
Letter Number Seq	11 (9,12)	11 (9,13)	10 (8,12)	NS
Judgment of line orientation	14 (12,15)	14 (12,15)	*13 (11,14)*	***p*** = 0.026*
Symbol digit modality	46 (39,53)	*43 (37,49)*	*37 (32,45)*	***p*** = 0.015*
Semantic fluency	52 (46,58)	51 (45,57)	50 (43,57)	NS
Letter fluency (F)	14 (11,17)	*13 (11,16)*	13 (11,16)	NS

Raw scores are presented. All rank order statistical evaluations have been adjusted for age, sex, and multiple comparisons. Differences between individuals who later develop progressive gait dysfunction (HY stage 3 or higher by 5 years of follow up) and those who maintain stable gait function under management are reported on the far right. Underline italic is used to denote differences between PD subgroups and controls. Bold italic denotes differences between PD subgroups (right column). Statistical comparisons are adjusted using a Bonferroni correction.

*p < 0.05 (Adjusted), **p < 0.005 (Adjusted).

### Development of emerging gait dysfunction scale factors

Logistic regression using all UPDRS items as regressors revealed 7 baseline UPDRS items that were associated at *p* < 0.01 with more rapid progression to HY stage 3 or higher within 5 years of disease diagnosis and were used to develop the ePIRS (Supplementary Table 1). These items consisted of 2 factors from UPDRS I (complaints of lightheadedness and fatigue), 3 factors from UPDRS II (self-perception of alterations in speech, walking, and ability to rise), and two factors from UPDRS 3 (objective findings of difficulty rising from a seated posture and visible stooping; Table 2). Two other items (difficulty turning in bed and adjusting clothes, from UPDRS section II, and pain and other sensations from UPDRS I), were significant at *p* < 0.05 after correction for multiple comparisons but were not included.

**Table 2:** Factors associated with progression to HY Stage 3 or worse within the first 5 years of disease.

Item (UPDRS Section)	PD Y5_HY0–2 Median (1st Q/3rd Q)	PD Y5_HY3–5 Median (1st Q/3rd Q)	Significance (univariate)	Multivariate logistical weight (rounded multiple of min)	Proposed weight	
					Present	Absent
Lightheadedness on standing (I)	0 (0,0)	0 (0,1)	*p* = 0.0042	0.91 (4)	4	0
Fatigue (I)	0 (0,1)	1 (0,1)	*p* = 0.0060	0.21 (1)	1	0
Speech (II)	0 (0,0)	0 (0,1)	*p* = 0.0023	0.85 (4)	4	0
Getting out of bed, car, or deep chair (II)	0 (0,1)	1 (0,1)	*p* = 0.00173	0.21 (1)	1	0
Walking and Balance (II)	0 (0,0.25)	1 (0,1)	*p* = 0.00051	0.74 (4)	4	0
Arising from chair (III)	0 (0,0)	0 (0,1)	*p* = 0.0055	1.22 (6)	6	0
Posture (III)	0 (0,1)	1 (0,1)	*p* = 0.0059	0.46 (2)	2	0

After correction for multiple comparisons (53 items were compared), 7 baseline UPDRS items were associated at *p* < 0.01 (after Bonferroni correction) with Y5_HY3–5, and were used to develop an early postural instability rating scale (ePIRS). Two other items (difficulty turning in bed and adjusting clothes, from UPDRS section II, and pain and other sensations from UPDRS I), were significant at *p* < 0.05 after multiple comparisons correction but were not included. Weights were derived from a logistical regression of items (present or absent) vs. status (PD Y5_HY3-5 vs. PD Y5_HY0-2).

### Development of ePIRS weighting

Quartile analysis revealed presence (1) or absence (0) of the UPDRS factor was a dominant characteristic at baseline. Specifically, median score, bottom quartile (25 percentile) score, and top quartile (75 percentile) score revealed scores universally between 0 and 1 at the quartiles. Therefore, to improve scale simplicity all items were binarized to present or absent, and a second logistic regression was performed, including all factors originally identified. Scale weighting for each factor was created by dividing individual weights of each item by the minimum weight of the lowest weighted item and rounding. Table 2 shows statistical properties of the group comparison, median and quartile properties by group, and weighting strategy.

### Properties of the ePIRS Scale

**Fig. 2:**
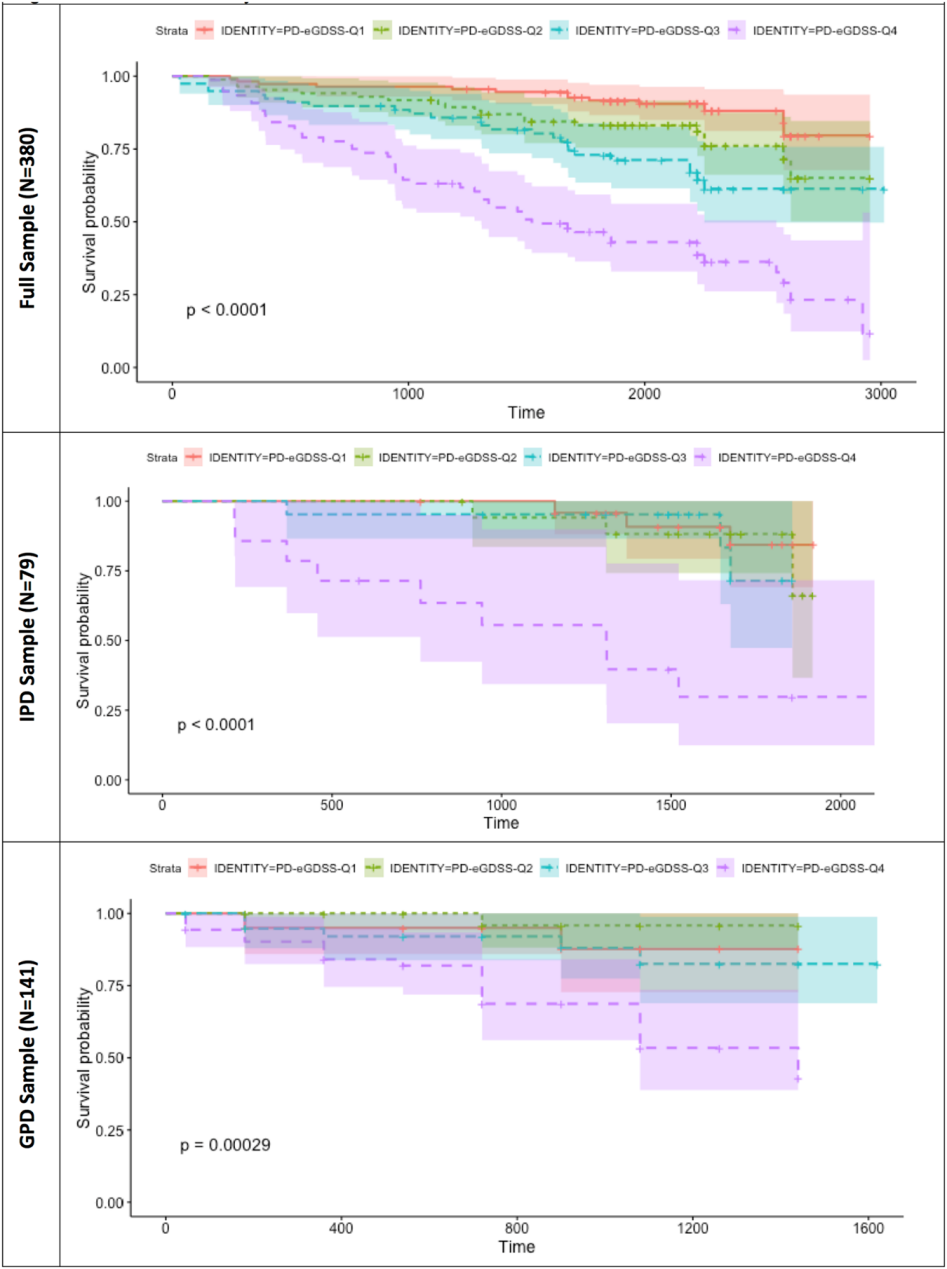
Survival plots by ePIRS quartile in PPMI. Properties of the ePIRS are shown in the full sample (Top) and in two validation sets. In both validation sets, membership in ePIRS quartile 4 is a significant predictor of later development of postural instability (HY 3) compared to both controls, and membership in Quartile I.

Properties of the ePIRS in the derivation sample (all 380 de-novo subjects) and both validation samples (79 de-novo subjects (dIPD group) and 141 subjects with genetic mutations associated with PD (vIPD group)) show that all individuals with PD, regardless of quartile, were (as expected) more likely to develop postural instability than controls—but quartile rank (and most specifically membership in quartile 4) was a substantial additional predictor. Table 3 shows Cox Proportional Hazard Ratio for development of disability by ePIRS Quartile in the full sample. Kaplan–Meier Survival Curves are shown in Fig. 2 in the derivation sample (Top), the IPD de-novo validation sample (Middle), and the GPD validation sample (Bottom). Quartile 4 (ePIRS score ≥ 11) is associated in both the derivation and validation sets, with a highly significant increased likelihood of developing HY stage 3.

**Table 3:** Baseline ePIRS quartile ranking predicts likelihood of advancing HY status.

	ePIRS quartile	Number in quartile	Quartile value (Score, Baseline)	Within group hazard ratio (HR) compared to PD quartile I (95% CI for HR)	*z*	Pr (>|*z*|)	Hazard ratio (HR) compared to controls (95% CI for HR)
	Controls	186	Median = 2	Controls I derivation reference group			
A: Derivation Sample (*N* = 380)	I	111	≤2				5.3 (1.7–16.3)***
	II	85	3–6	2.03 (1.00–4.11)	1.97	0.049	10.7 (3.7–31.5)****
	III	78	7–10	3.34 (1.71–6.54)***	3.52	0.00043	17.7 (6.2–51.0)****
	IV	76	≥11	8.28 (4.48–15.29)****	6.75	<0.00001	44.1 (15.9–122.2)****
B: IPD Sample (*N* = 79)	I (Ref)	86	Derivation sample de-novo quartile I reference group (Modified)				
	I	25	≤2	1.90 (0.51–7.02)	0.96	0.34	9.7 (1.9–48.4)**
	II	18	3–6	2.52 (0.68–9.29)	1.38	0.17	12.7 (2.6–63.6)***
	III	21	7–10	2.53 (0.68–9.47)	1.38	0.17	13.2 (2.6–67.3)***
	IV	14	≥11	16.18 (6.34–41.23)****	5.83	<0.00001	88.0 (23.2–333.5)****
C: GPD Sample (*N* = 141)	I (Ref)	111	Derivation sample de-novo quartile I reference group				5.3 (1.7–16.3)***
	I	20	≤2	2.81 (0.56–14.06)	1.26	0.21	21.6 (2.5–185.3)***
	II	29	3–6	0.94 (0.11–7.87)	−0.056	0.96	7.4 (0.6–95.1)
	III	43	7–10	3.84 (1.15–12.83)	2.19	0.029	28.5 (4.7–174.1)***
	IV	60	≥11	13.26 (5.13–34.28)****	5.34	<0.00001	103.2 (18.7–569.9)****

Progression to HY ≥ 3, by quartile, compared to healthy controls (right-most columns) and compared to Quartile I. Reference Quartile I values in all cases are taken from the de-novo sample. A: Significant, and progressive impact of quartile on risk development of HY ≥ 3 is noted in the de-novo PD derivation sample. B: In the IPD validation sample, ePIRS scores in the fourth quartile (≥11) were associated with a significantly higher likelihood of progression to HY ≥ 3. C: In the GPD cohort, once again membership in Quartile 4 confers a higher risk of progression to HY ≥ 3 compared to de-novo subjects with ePIRS scores ≤ 2.

**p ≤ 0.01, ***p ≤ 0.005, ****p ≤ 0.0001.

We also evaluated the efficacy of ePI phenotype as a prognostic indicator for development of postural instability. We examined the temporal relationship between development of the ePI phenotype (ePIRS score ≥ 11) and development of postural instability (HY ≥ 3) in both the IPD and the GPD cohorts. Any instance of either ePI phenotype score 11, or HY 3, was considered a potential paired ePI-HY “event”, in the full sample of 380 individuals. We therefore had 301 ePI “events” (79 individuals had neither occurrence). In the IPD cohort, ePIRS Score 11 occurred prior to HY 3, or HY 3 had not yet occurred by end of follow up, in 289 of 301 pairings. HY 3 occurred before ePIRS 11 (or ePIRS 11 had not occurred at time of end of follow up) in 12 cases (chi-square statistic = 510, *p* < 0.00000001). Median time from ePIRS Score 11 to HY 3 in those who developed postural instability was 983 days.

### Relationship of ePIRS score to cognitive decline in PD

As we observed that ePIRS Quartile 4 substantially deviates from other quartiles in both derivation and validation analyses, we evaluated the relationship between membership in Quartile 4 and slope of cognitive change, comparing ePIRS Quartile 4 to Quartile 1. At baseline the median Montreal Cognitive Assessment (MOCA) score for quartile 1 and 2 was 28 (interquartile range (IQR) for quartile 1 and 2 = 27–29, and for quartiles 3 and 4 was 27, with an IQR for quartile 3 of 26–29 and for quartile 4 of 26–28. The median MOCA score for healthy controls was also 28, with an IQR of 27–29. The difference in MOCA score between quartile 1 and 4 was not statistically significant *t* = 2.52 (1, 166) *p* = 0.13. However, membership in Quartile 4 conferred a significant increased likelihood of cognitive progression, as measured by the MOCA and Hopkins Verbal Learning Test (HVLT), after correction for multiple comparisons. Rate of change in total UPDRS score in de-novo subjects was not different across ePIRS score quartile, and rate of cognitive change did not differ significantly between Quartiles 1–3. An additional analysis of rate of UPDRS score change including Quartiles 1–3 versus Quartile 4 resulted in similar results to those shown in Table 4.

**Table 4:** Change in motor, neurobehavioral, and cognitive function, slope (mean point change per year), with 95% confidence intervals, for motor and cognitive scores in PPMI for all de-novo subjects with at least 5 years of data available.

Measure	Controls	Parkinson disease		Chi-square (LR) (ePIRS Q1 vs. Q4)
	*N* = 185 (mean + /− 95CI)	*N* = 76 ePIRS Quartile 4 (Mean + /− 95CI)	*N* = 111 ePIRS Quartile 1 (Mean + /− 95CI)	
*Motor examination*				
UPDRS I	0.10 ± 0.10	0.61 ± 0.22***	0.74 ± 0.12***	0.120
UPDRS II	0.049 ± 0.044	1.44 ± 0.34***	1.09 ± 0.22***	0.419
UPDRS III	−2.0 ± 0.85	1.34 ± 0.72***	0.92 ± 0.43***	0.637
*Cognition*				
MOCA	0.02 ± 0.12	−0.70 ± 0.23***	−0.19 ± 0.12***	0.002**
HVLT Total Recall	0.12 ± 0.16	−0.63 ± 0.25***	0.12 ± 0.28	0.008**
Letter-number sequencing	0.01 ± 0.11	−0.22 ± 0.18***	0.07 ± 0.11	0.145
Judgment of line orientation	0.01 ± 0.09	−0.25 ± 0.14***	−0.01 ± 0.12	0.193
Symbol digit modality	0.34 ± 0.43	−1.91 ± 0.64***	−0.59 ± 0.59***	0.092
Semantic fluency	−0.05 ± 0.51	−1.33 ± 0.65***	−0.21 ± 0.67*	0.157

Significance is adjusted for age and sex; for cognitive measures significance of 0.00833 (0.05/6) is required to achieve statistical threshold.

*p < 0.05, **p < 0.00833, ***p < 0.0001.

### Relationship of ePIRS score to year 5 PIGD score

We also examined the relationship between ePIRS score and development of the broader PIGD phenotype over the 5-year study period, and contrasted performance with traditional UPDRS measures in the de novo validation set. While age, gender, and UPDRS 1, 2, and 3 scale total scores (5 factors) accounted for 19.9% of variance in predicting Y5-PIGD, the proposed ePIRS score (3 factors age, gender, and ePIRS) accounted for an adjusted 27.4% of variance (*p* = 0.015; Table 5). This difference is not surprising because the UPDRS score is heavily influenced by tremor and those with tremor dominant presentation tend to have slower rate of progression^26^. In contrast, the ePIRS is based on axial signs (lightheadedness, alterations in speech, walking, ability to rise and visible stooping). In summary, ePIRS score at baseline explained more variance in Y5_PIGD than UPDRS, and UPDRS did not add significantly to the ePIRS score with regards to explaining variance in Y5_PIGD.

**Table 5:** Relative relationship of ePI to PIGD phenotype.

Source	*F*	Pr > *F*	SOURCE	*F*	Pr > *F*
AGE	8.282	0.005	ePIRS	15.999	<0.00001
UPDRS I	0.753	0.388	AGE	5.354	0.023
UPDRS II	2.040	0.158	Sex	0.000	0.998
UPDRS III	3.059	0.085			
Gender	0.177	0.675			
(adjusted) Explained variance		0.199	(adjusted) Explained variance		0.274**

While age is superior to the UPDRS in predicting progression, the baseline ePIRS score is superior to age in the de-novo validation set (*N* = 79).

**p = 0.015.

### Post-hoc analyses—capacity of baseline PIGD scale to predict year 5 PIGD and H&Y scale score

We also compared our new metric to the existing metrics by determining if baseline PIGD scale score provided relevant information to predict later HY status and PIGD scores. Baseline PIGD, which is typically low, was a poor predictor of Year 5 PIGD score in both the derivation (3 factors, adjusted *R*^2^ = 0.172) and de-novo validation set (adjusted *R*^2^ = 0.169), and did not improve prediction of Year 5 PIGD when added to the UPDRS (*R*^2^ = 0.254, similar to UPDRS alone). In a 4 factor ANOVA model, baseline PIGD (*F* statistic = 0.011, Pr > *F* = 0.917), did not improve prediction when added to the proposed ePIRS scale, and adjusted estimated accounted variance (25.1%) declined due to addition of an additional factor. In a logistic model (prediction of Y5_HY3–5), adding PIGD to ePIRS (4 factors, area under ROC curve = 0.784) did not improve prediction of the 3-factor model (Age, Gender, and ePIRS score alone, area under ROC curve = 0.784).

## Discussion

We describe a link between measurable, baseline clinical symptoms in PD in those without postural instability, and development of postural instability. We have shown that specific clinical features predict development of postural instability in PD years prior to the onset of these disabling signs, allowing us to identify individuals at high risk for rapid disease progression. The proposed ePI phenotype was robust in two separate validation sets: (1) a de-novo set of individuals with idiopathic PD and (2) a validation set of individuals with variable time of onset of symptoms with one of three known genetic causes of PD. Based on these findings, we propose an emerging postural instability phenotype that can be detected in de novo patients that is associated with high risk of accelerated development of both motor and cognitive dysfunction.

We operationalize detection of the ePI phenotype using a provisional emerging postural instability rating scale (Supplementary Table 1). Significant postural instability in de-novo, early PD is uncommon, and in fact is considered a criterion for questioning a diagnosis of PD and considering other causes of parkinsonism^35^. Not surprisingly, therefore, none of the de-novo subjects in the PPMI dataset had postural instability at baseline. In the genetic cohort we used for one of our validation sets, we excluded those with HY of 3 or above from our evaluation cohort, limiting the subject pool to those with no or minimal postural instability at baseline by Hoehn and Yahr criteria. The identification of ePI phenotype using our scale preceded development of postural instability by a median of 2–3 years.

As with our postural instability measure, UPDRS derived gait scores have been shown to be associated with steeper motor and cognitive decline^36^, however not all studies agree^37–43^. For example, Zeighami and colleagues^44^ found that the MDS-UPDRS derived gait score was not predictive of disease progression. In addition, Lee and colleagues^45^ classified 325 de-novo PD patients from the PPMI database into tremor dominant, PIGD and indeterminant groups. At study onset, dopamine transporter uptake was highest in the tremor dominant group, however after 4 years of follow up there were no differences in UPDRS III motor scores, MOCA based cognitive function or dopaminergic innervation between the tremor dominant and PIGD groups. This work is consistent with the idea that disease progression is a significant contributor to the relationship between PIGD and tremor dominant disease and may also subserve differences in study outcomes. Furthermore, PD genetic variants have distinct phenotypes. For example, the APOE 4 allele has been associated with increased cognitive dysfunction in PD^46^. GBA variants have been linked to more severe symptoms including cognitive and motor outcomes compared to G2019s-LRRK2^47^. In contrast, LRRK2 rs34637584 minor allele carriers showed reduced cognitive dysfunction compared to GBA variants. These genetic differences add variability to research samples and could also account for differences in findings across studies. However, the proposed ePIRS membership in Quartile 4 did predict decline to postural instability in our genetic LRRK2 + GBA sample. Outcome differences across studies may also be due to the fact that UPDRS based scales like the ePIRS have an acknowledged weakness in relying significantly on patient self-reports. Furthermore, symptomatic interventions complicate interpretation of performance-based measures, though there is evidence that scales like the UPDRS reflect the long term impact of PD, rather than short term fluctuations in performance^48^. Thus, this initial version of the ePIRS may be useful in and of itself as a simple screening tool and may also provide guidance in developing an objective baseline evaluation to more clearly define the phenotype.

While more detailed studies to develop ideal cutoff scores would be appropriate, at this stage our data suggest that an ePIRS score ≥ 11 (indicating quartile 4) is a reasonable criterion for identifying the phenotype. Individuals in the 4th quartile for ePIRS score had substantially increased risk of developing disabling postural instability as measured by the H & Y rating scale, and showed accelerated cognitive progression as measured by the MOCA and HVLT. Our findings are consistent with previous work showing a link between PIGD and cognitive dysfunction in early PD. For example, Lord and colleagues^49^ demonstrated an association among measures of executive function and attention, and gait parameters including pace, rhythm, variability and postural control in subjects with an HY score ≥ 2.

From a practical perspective, a rapid clinical evaluation that can define future risk of disability is particularly useful. Heretofore, the UPDRS has been a primary target in intervention studies. However summary UPDRS metrics are primarily useful within subjects for comparing the impact of dopaminergic responsive motor symptoms, and are less useful for evaluating the impact of potential disease modifying therapies^3^. We show here that for the majority of individuals in the first 5 years of observation in PPMI (the first 6 years of diagnosed disease), changes in postural instability under management are modest, but progressive in a sub-population despite treatment. By formally identifying this emerging postural instability phenotype, we define a population at relatively high risk for dopamine resistant symptoms that can be targeted for early intervention studies. However it is important to consider that the link between resistance to dopamine replacement and PIGD is complex. Lack of a robust response to dopaminergic medication is also associated with disease progression, motility disorders, absorption changes and medication side effects.

The clinical features we identify in this work are consistent with specific pathophysiology of gait dysfunction in PD. We found the following features appear to be associated with more rapid progression of gait dysfunction: (1) lightheadedness on standing, (2) self-perceived speech difficulties, (3) difficulties standing/rising and (4) postural changes. While gait and posture factor prominently in the ePI phenotype, the characteristics are heterogeneous. One point in common among all of the characteristics defined by the ePIRS is that the symptoms all represent signs associated with abnormalities in distributed brainstem networks. For example, changes in standing/arising, and postural changes could be related to alterations in the substantia nigra and mesencephalic gait center^36,50,51^. Lightheadedness on standing is consistent with abnormalities in adrenergic and noradrenergic outflow, suggesting either a peripheral or central (brainstem) lesion in noradrenergic and adrenergic neurons^52^. Self-perceived changes in speech could be an early cognitive finding, but also a lesion in the dorsal motor nucleus of the vagus (one of the first regions impacted by Lewy bodies in PD in Braak’s formulation)^29,53,54^, or more distributed brainstem pathology. In all cases, the common thread may be a more aggressive progression of synucleinopathy in the brainstem. The association of these apparently diffusely distributed brainstem-related symptoms with more rapid cognitive deterioration suggests a more aggressive overall disease process.

In summary, we show that risk for developing postural instability and rapid disease progression is detectable at baseline clinically, based on symptoms and clinical findings (the ePI phenotype) that are distinct from those that later characterize the PIGD phenotype. It is important to note that many factors could account for accelerated progression that were not available in the PPMI database. Furthermore, we did not examine the contribution of dopamine replacement therapy to ePI progression. Identifying individuals at high risk of clinical progression is key to sample homogeneity for the study of pathophysiology of PD progression and clinical intervention. These interventions could include changes in medication strategy, physical therapy or even putative neuroprotective drugs. These findings are consistent with the observation that severity of motor symptoms in PD, and rate of change of these symptoms over time is quite variable, suggesting the existence of disease subgroups with varying rates of progression^35,55–59^. Amongst this variability, individuals with PD universally share symptomatic dopamine deficiency, which has led to a wave of innovation to assist with management of dopaminergic-related symptoms. However, treating PD as a monomorphic condition has, as noted by Espay and colleagues, “consistently failed when testing potential disease-modifying interventions”^60^. We demonstrate that a variety of detectable signs and symptoms already measured in the UPDRS predict rapid disease progression. These findings identify individuals with PD in which neuroprotective intervention is needed, and in which results of an intervention are potentially measurable within the confines of a clinical trial.

## Methods

### Selection of sample

Data were downloaded from the Parkinson’s Progressive Markers Initiative (PPMI)^61^ in January of 2019. The study was approved by the institutional review board of the University of Alabama at Birmingham. Individual subjects provided informed consent under institutions participating in PPMI data collection. PPMI participants received study visits at least annually. We screened for data sets that contained at least 5 years of clinical data and identified 380 individuals with de-novo idiopathic PD in PPMI who met our criteria. The inclusion criteria for this de-novo cohort was PD diagnosis within 1 year and symptom onset within 2 years, no dopaminergic treatment, and HY stage 2 or better. A derivation idiopathic PD (dIPD) set of 301, and a validation idiopathic PD (vIPD) set of 79 were developed. The vIPD sample was developed based on availability of imaging, to allow for ancillary analysis and further study. For additional validation, we selected the PPMI genetic cohort. All individuals in this cohort had mutations in the synuclein alpha (SNCA), leucine-rich repeat kinase 2 (LRRK2), or glucocerebrosidase 1 (GBA1) gene. The genetic PD (GPDv) cohort at the time of this analysis contained 220 enrolled individuals. PD inclusion criteria in the genetic cohort differed from the IPD cohort, and included PD diagnosis for <7 years, and HY < 4 at entry. In this genetic cohort we restricted our sample to individuals with HY status 2 or better (no balance disturbance). Within the cohort, 141 individuals met inclusion criteria. As a comparative sample, we identified 183 healthy controls who similarly had at least 5 years of clinical follow up. All individuals in PPMI with PD have an “on medication” evaluation at each visit. Thus, we studied dIPD = 301, vIPD = 79, GPDv = 141, Controls = 183.

### Sample features

Age and sex data were available in PPMI and included in our analysis. Motor assessments included the modified Unified Parkinson’s Disease Rating Scale, and HY rating. From the UPDRS, we calculated PIGD score at each time point over a minimum of 5 years. Cognitive measures included the Montreal Cognitive Assessment (MOCA), the Hopkins Verbal Learning Test (HVLT), Letter Number Sequencing (LNS), Judgment of Line Orientation (JLO), and Symbol-Digit Modality (SDM). All evaluations were performed at baseline and at multiple time points, allowing us to calculate both baseline differences and rate of change by group. Slope of cognitive change in this sample was expressed by change in mean score value divided by time in years. For demographic data, 2-sample t-tests were used to evaluate group differences using XLstat (https://www.xlstat.com/en/). All group comparisons were two-tailed and corrected for multiple comparisons using a Bonferroni correction where appropriate.

### Scale development and sample selection

Hoehn and Yahr Stage (HY)^1,2^ was dichotomized, with a score of 3 or higher indicating clinically detectable postural instability during stance, in the derivation set (301 subjects) to categorize individuals who by year 5 had developed gait dysfunction (*n* = 85 Y5_HY3–5) and those who had not (*n* = 216 Y5_HY0–2). We classified a subject as Y5_HY3–5 if at any time over the first 5 years a clinical rating of HY 3 was scored by any rater while “on-medication”. The classification of Y5_HY0–2 was given to any individual who was never classified by any rater as above HY stage 2. We also measured PIGD using the PIGD score derived by Jankovic and colleagues at each follow up visit^3,4^. Average year 5 PIGD scores were also derived from “on-medication” evaluations in years 4.5–6 averaged over 2–3 visits to calculate a year 5 outcome variable (Y5_PIGD).

We developed a scale that was designed to capture items in the UPDRS associated with disability (onset of postural instability, HY 3 or greater). Specifically, the Hoehn and Yahr status of visits 8–11 in the sample (approximately 4.5 to 6 years) were evaluated; if any one of the visits showed HY stage 3 (postural instability), the subject was identified as an individual who had achieved this progression milestone. Individuals who had Hoehn and Yahr stage of 2 or less for all three visits were characterized as not achieving this milestone. A logistic regression was performed where individual elements on the Unified Parkinson’s Disease Rating Scale (sections 1 through 3) from the baseline visit were regressed against this binary logistic factor (achieved or did not achieve HY stage 3). A Bonferroni correction was used and items with an adjusted p value of less than 0.01 (after correction) were selected to generate the predictive model. The sample involved 53 multiple comparisons and analyses were corrected accordingly for multiple comparisons. Note the HY-based classification neatly dichotomizes our sample into two groups that, during treatment (“on-medication”), were either with or without substantial progression of gait dysfunction, over the first 5 years of disease (Fig. 1). For the selected items, weights were generated based on rounding of an idealized model (see Table 2). The adjusted score was then used to generate quartile-level cutoff values for the sample, from lowest (ePIRS score 0–2) to highest (ePIRS score 11 or above).

### Scale validation

EPI quartiles were generated based on ePIRS score and subjected to survival or time-to -event analysis where the event was defined as conversion to H & Y scale score of 3 or more. We compare performance across quartiles calculated based on median values of (1) ePIRS score at year 5, and (2) in a separate analysis on HY scale score at year 5. We performed a survival analysis to evaluate the relationship between scale value (quartile rank) and population time to progression of gait related disability (at least one practitioner evaluation of HY 3) in both validation sets, using a Cox proportional hazard regression model. Survival plots for ePIRS score quartiles were calculated using R Studio version 3.4, including hazard ratios, *z* scores, and probability scores (Fig. 2). The time-to event analysis is presented in the scale derivation sample, and in two validation samples which were not used for scale verification. Change in motor and cognitive performance from baseline (year 0) to year 5 were derived for individuals with de-novo PD and controls, for individuals in quartile 1 (the slowest progressing quartile by gait criteria) versus individuals in quartile 4 (the most rapidly progressive quartile by gait criteria) and compared using a 2-sample *t*-test.

Logistic regression and receiver operating characteristic curve analyses were used to compare the utility of a 5-factor model (age, gender, and UPDRS items I, II, and III) compared to a 3-factor model (age, gender, and ePIRS) to predict progression (Y5_PIGD). All critical *p* values underwent Bonferroni correction for multiple comparisons.

Finally, we performed a regression analysis to compare PIGD score at approximately year 5 to ePIRS score, age, and sex at baseline. PIGD scores were taken from visits 8–11 and averaged. The regression analysis presents F-statistics for the model (3 degrees of freedom) and p values for each independent element of the model (age, gender, and ePIRS score). This model is compared to segments 1, 2, and 3 of the UPDRS, as well as age and gender (5 degrees of freedom). Note, the UPDRS has a 4th element (motor complications of therapy); this portion of the model was not measured in the vast majority of de-novo subjects at baseline in PPMI as the subjects at this point were not on therapy that could cause motor fluctuations; the 4th segment of the UPDRS was therefore excluded from our analysis.

### Reporting summary

Further information on research design is available in the Nature Research Reporting Summary linked to this article.

## Supplementary information

Supplementary Table 1

Reporting Summary

## Data Availability

All data are publicly available from PPMI upon request (https://www.ppmi-info.org/access-data-specimens/download-data/).
