# Supplementary material for: The emerging postural instability phenotype in idiopathic Parkinson disease
Source: NPJ Parkinsons Dis. 2022 Mar 18;8:28. doi: 10.1038/s41531-022-00287-x (PMC8933561; doi:10.1038/s41531-022-00287-x)
Supplement: Supplementary file 1 — Supplementary Table 1 [file 41531_2022_287_MOESM1_ESM.pdf]

Emerging Postural Instability Rating Scale				
			Points	Score
1	Lighthheadedness on Standing	yes ☐	4	☐
		no ☐	0	
2	Fatigue	yes ☐	1	☐
		no ☐	0	
3	Speech Difficulties	yes ☐	4	☐
		no ☐	0	
4	Difficulty getting out of bed, car or deep chair	yes ☐	1	☐
		no ☐	0	
5	Difficulty with walking and balance	yes ☐	4	☐
		no ☐	0	
6	Difficulty arising from chair	yes ☐	6	☐
		no ☐	0	
7	Stooped posture	yes ☐	2	☐
		no ☐	0	
	**Total Score** ☐ ePIRS ≥ 11			

Supplementary Table 1. The emerging postural instability rating scale contains 7 yes/no items associated with weighted scores. A score ≥ 11 indicates ePIRS phenotype.
