# Supplementary material for: The emerging postural instability phenotype in idiopathic Parkinson disease
Source: NPJ Parkinsons Dis. 2022 Mar 18;8:28. doi: 10.1038/s41531-022-00287-x (PMC8933561; doi:10.1038/s41531-022-00287-x)
Supplement: Supplementary file 2 — Reporting Summary [file 41531_2022_287_MOESM2_ESM.pdf]

nature research

Corresponding author(s): Elizabeth Disbrow
edisbr@lsuhsc.edu

Last updated by author(s): Aug 19, 2020

Reporting Summary

Nature Research wishes to improve the reproducibility of the work that we publish. This form provides structure for consistency and transparency in reporting. For further information on Nature Research policies, see our Editorial Policies and the Editorial Policy Checklist.

Statistics

For all statistical analyses, confirm that the following items are present in the figure legend, table legend, main text, or Methods section.

n/a	Confirmed	
☐	☒	The exact sample size (*n*) for each experimental group/condition, given as a discrete number and unit of measurement
☐	☒	A statement on whether measurements were taken from distinct samples or whether the same sample was measured repeatedly
☐	☒	The statistical test(s) used AND whether they are one- or two-sided<br>*Only common tests should be described solely by name; describe more complex techniques in the Methods section.*
☐	☒	A description of all covariates tested
☐	☒	A description of any assumptions or corrections, such as tests of normality and adjustment for multiple comparisons
☐	☒	A full description of the statistical parameters including central tendency (e.g. means) or other basic estimates (e.g. regression coefficient) AND variation (e.g. standard deviation) or associated estimates of uncertainty (e.g. confidence intervals)
☐	☒	For null hypothesis testing, the test statistic (e.g. *F*, *t*, *r*) with confidence intervals, effect sizes, degrees of freedom and *P* value noted<br>*Give P values as exact values whenever suitable.*
☒	☐	For Bayesian analysis, information on the choice of priors and Markov chain Monte Carlo settings
☒	☐	For hierarchical and complex designs, identification of the appropriate level for tests and full reporting of outcomes
☒	☐	Estimates of effect sizes (e.g. Cohen's *d*, Pearson's *r*), indicating how they were calculated

Our web collection on statistics for biologists contains articles on many of the points above.

Software and code

Policy information about availability of computer code

Data collection	Data were downloaded from the Parkinson's Progressive Markers Initiative (PPMI)44 in January of 2019.
Data analysis	Survival plots for eGD quartiles were calculated using R Studio version 3.4, including hazard ratios, z scores, and probability scores.<br>Analysis of variance for group comparisons and a logistic regression examining the relationship between eGD phenotype and development of PIGD characteristics were was performed using XLstat, which included F statistics and probability information.

For manuscripts utilizing custom algorithms or software that are central to the research but not yet described in published literature, software must be made available to editors and reviewers. We strongly encourage code deposition in a community repository (e.g. GitHub). See the Nature Research guidelines for submitting code & software for further information.

Data

Policy information about availability of data

All manuscripts must include a data availability statement. This statement should provide the following information, where applicable:

- Accession codes, unique identifiers, or web links for publicly available datasets
- A list of figures that have associated raw data
- A description of any restrictions on data availability

All data are publicly available from PPMI upon request (https://www.ppmi-info.org/access-data-specimens/download-data/).

Field-specific reporting

Please select the one below that is the best fit for your research. If you are not sure, read the appropriate sections before making your selection.

☒ Life sciences ☐ Behavioural & social sciences ☐ Ecological, evolutionary & environmental sciences

For a reference copy of the document with all sections, see nature.com/documents/nr-reporting-summary-flat.pdf

Life sciences study design

All studies must disclose on these points even when the disclosure is negative.

Sample size	We screened for data sets that contained at least 5 years of clinical data and identified 380 individuals with de-novo idiopathic PD in PPMI who met our criteria. The inclusion criteria for this de-novo cohort was PD diagnosis within 1 year and symptom onset within 2 years, no dopaminergic treatment, and HY stage 2 or better. A derivation idiopathic PD (dIPD) set of 301 was identified. As a comparative sample, we identified 183 healthy controls who similarly had at least 5 years of clinical follow up.
Data exclusions	Because the goal of the project was to predict conversion to PIGD (postural instability and gait disturbance), subjects presenting with PIGD, defined as an H & Y scale score of 3 or more, was excluded from the analysis.
Replication	For model validation 2 held out samples were identified. A validation idiopathic PD (vIPD) set of 79 were developed that was based on availability of imaging, to allow for ancillary analysis and further study. For additional validation, we selected the PPMI genetic cohort. All individuals in this cohort had mutations in the synuclein alpha (SNCA), leucine-rich repeat kinase 2 (LRRK2), or glucocerebrosidase 1 (GBA1) gene. The genetic PD (GPDv) cohort at the time of this analysis contained 220 enrolled individuals. PD inclusion criteria in the genetic cohort differed from the IPD cohort, and included of PD diagnosis for < 7 years, and HY < 4 at entry. In this genetic cohort we restricted our sample to individuals with HY status 2 or better (no gait disturbance). Within the cohort, 141 individuals met inclusion criteria.
Randomization	Subjects grouping was based on disease group, including idiopathic PD and genetic cohort, as well as availibility of brain imaging data (held out IPD group).
Blinding	Analysis was performed on an existing database. The bulk of the analysis was performed within a single group. Group membership is not a key factor in the outcome of the study.

Reporting for specific materials, systems and methods

We require information from authors about some types of materials, experimental systems and methods used in many studies. Here, indicate whether each material, system or method listed is relevant to your study. If you are not sure if a list item applies to your research, read the appropriate section before selecting a response.

Materials & experimental systems

n/a	Involved in the study	
☒	☐	Antibodies
☒	☐	Eukaryotic cell lines
☒	☐	Palaeontology and archaeology
☒	☐	Animals and other organisms
☐	☒	Human research participants
☒	☐	Clinical data
☒	☐	Dual use research of concern

Methods

n/a	Involved in the study	
☒	☐	ChIP-seq
☒	☐	Flow cytometry
☒	☐	MRI-based neuroimaging

Human research participants

Policy information about studies involving human research participants

Population characteristics	Demographic data are reported in Table 1. Age was used as a covariate where appropriate because the mean age of the PIGD group was greater than the non-PIGD and control group.
Recruitment	The authors did not participate in subject recruitment. Data was downloaded from the PPMI database, which contains behavioral and brain imaging data from multiple sites.
Ethics oversight	The study was approved by the institutional review board of the University of Alabama at Birmingham.

Note that full information on the approval of the study protocol must also be provided in the manuscript.
